# CDKL5 deficiency disorder: molecular insights and mechanisms of pathogenicity to fast-track therapeutic development

**DOI:** 10.1042/BST20220791

**Published:** 2022-08-23

**Authors:** Nicole J. Van Bergen, Sean Massey, Anita Quigley, Ben Rollo, Alexander R. Harris, Robert M.I. Kapsa, John Christodoulou

**Affiliations:** 1 Brain and Mitochondrial Research Group, Murdoch Children's Research Institute, Royal Children's Hospital, Melbourne, Australia; 2 Department of Paediatrics, University of Melbourne, Melbourne, Australia; 3 Electrical and Biomedical Engineering, School of Engineering, RMIT University, Melbourne, VIC, Australia; 4 Aikenhead Centre for Medical Discovery, St Vincent's Hospital Melbourne, Fitzroy, Melbourne, VIC 3065, Australia; 5 Centre for Clinical Neurosciences and Neurological Research, St. Vincent's Hospital Melbourne, Fitzroy, VIC 3065, Australia; 6 Department of Medicine, St Vincent's Hospital Melbourne, The University of Melbourne, Fitzroy, Melbourne, VIC 3065, Australia; 7 Aikenhead Centre for Medical Discovery, Department of Biomedical Engineering, University of Melbourne, Melbourne 3010, Australia; 8 Department of Neuroscience, Central Clinical School, Monash University, Melbourne, Australia; 9 Victorian Clinical Genetics Services, Murdoch Children's Research Institute, Melbourne, VIC, Australia; 10 Discipline of Child and Adolescent Health, University of Sydney, Sydney, Australia

**Keywords:** CDKL5 deficiency disorder, kinase, neurodevelopmental disorders, phosphorylation

## Abstract

CDKL5 deficiency disorder (CDD) is an X-linked brain disorder of young children and is caused by pathogenic variants in the *cyclin-dependent kinase-like 5* (*CDKL5*) gene. Individuals with CDD suffer infantile onset, drug-resistant seizures, severe neurodevelopmental impairment and profound lifelong disability. The CDKL5 protein is a kinase that regulates key phosphorylation events vital to the development of the complex neuronal network of the brain. Pathogenic variants identified in patients may either result in loss of CDKL5 catalytic activity or are hypomorphic leading to partial loss of function. Whilst the progressive nature of CDD provides an excellent opportunity for disease intervention, we cannot develop effective therapeutics without in-depth knowledge of CDKL5 function in human neurons. In this mini review, we summarize new findings on the function of CDKL5. These include CDKL5 phosphorylation targets and the consequence of disruptions on signaling pathways in the human brain. This new knowledge of CDKL5 biology may be leveraged to advance targeted drug discovery and rapid development of treatments for CDD. Continued development of effective humanized models will further propel our understanding of CDD biology and may permit the development and testing of therapies that will significantly alter CDD disease trajectory in young children.

## CDKL5 deficiency disorder (CDD) clinical phenotype and genetics

CDKL5 deficiency disorder (CDD) is a neurodevelopmental encephalopathy, characterized by early onset, refractory seizures and gross developmental delay that affects up to 1 : 40 000 live births [1]. *Cyclin-dependent kinase-like 5* (OMIM:300203, 300672) was identified as the disease-causing gene in 2004 [2,3]. CDD was initially considered an early onset seizure variant of Rett syndrome (RTT) [4], but is now considered an independent disorder [5,6]. Despite CDD being rare, pathogenic variants in *CDKL5* are among the most common genetic causes of severe epilepsy in childhood and the underlying cause of a spectrum of milder clinical phenotypes [7,8].

Discovered during mapping of the X chromosome [9], *CDKL5* is located on the Xp22.13 cytogenetic band and is subject to random X-inactivation in females [10]. There are hundreds of known pathogenic mutations in *CDKL5* [2–4,8,10,11] and most mutations are *de novo* [8], resulting in impaired kinase activity [12]*.* Recent reviews provide an excellent summary [13–15]. A small proportion of CDD patients are mosaic, predominantly males [16], normally assessed from peripheral blood, but this may not reflect the X-chromosome inactivation (XCI) pattern in the brain [17,18] which may have profound effects on clinical severity. X-linked mosaicism of *Cdkl5* deficiency may underly spontaneous seizures in female CDD mice [19]. Mosaicism is common in other genetic neurological disorders, and the degree of mosaicism may be higher than previously estimated for CDD.

Individuals with CDD experience seizures generally commencing by 12 months [1,3,5,20–23], 90% suffer onset by 3 months [24], and only 12% remain seizure-free for >12 months [25,26]. Seizures range from tonic, tonic-clonic and myoclonic spasms, or seizures with no clear pattern. Seizures initially respond to a range of broad-spectrum anti-epileptic treatments [27] but often return for life and may be in a different form [20,21]. Other seizure modulators used include cannabis derivatives and ketogenic diets, but their effectiveness in reducing seizures is limited and wanes over time [27]. Seizure frequency, the level of functional impairment and the anti-seizure medication regimen all influence quality of life for CDD individuals [28]. Adequate seizure control can improve developmental outcomes [29], particularly when implemented early [30] leading to reduced cost to both individual and society. Since CDD was identified in 2004, it is unclear what the average lifespan is, but the oldest patients reported are in their 40s.

CDD patients experience long episodes without sleep, called ‘all night parties’ [1], may night-walk [5,23,26,31] and frequently have respiratory, musculoskeletal and gastrointestinal problems [23]. Gross motor functions and hand functions such as grasping, are delayed or never achieved [32], and few master finer motor movements [26,33]. Males are less likely to achieve developmental milestones [26,33], which may be a consequence of having only one copy of the X chromosome carrying a pathogenic *CDKL5* allele. Although one study found no significant sex difference in milestone achievement, their analysis did not exclude known mosaic patients [34], which may contribute to the clinical complexity of CDD [16] warranting further investigation. New assessment tools are gathering a more accurate natural history of CDD [34]. Recent advances in stem cell modeling and development of human iPSC-based models allow for the creation of relevant human tissue models to reveal new kinase targets, for understanding molecular function, development, functional activity, drug efficacy and the tailoring of personalized medicine [27,35–37].

## CDKL5 protein structure

CDKL5 is a serine/threonine protein kinase of the CMGC (cyclin-dependent kinases (CDKs), mitogen-activated protein kinases (MAPKs), glycogen synthase kinases (GSKs) and Cdc2-like kinases (CLKs)) kinase group, all share a high degree of sequence homology in the kinase domain. The CMGC kinases are linked to transcription, RNA processing, cellular communication and regulation of the cell cycle, but there are many undiscovered functions of individual kinases. The crystal structure of the human CDKL family revealed structural divergence from the other CD- and MAP-kinases, and the CDKL family have a distinct function in cilial regulation [38]. Some CMGC members form physical interactions with other proteins, but CDKL kinases have a limited number of known binding partners [39].

**Figure 1.**
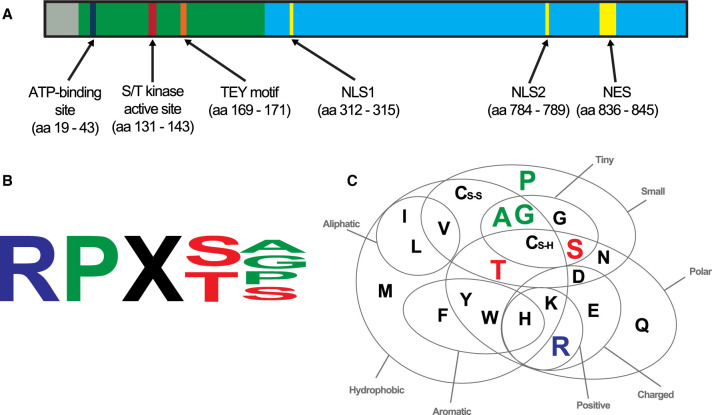
CDKL5 protein domains consensus sequence in phosphorylation targets. (**A**) The N-terminus harbors the sequences relevant to the kinase catalytic function within the kinase domain (green), including the ATP-binding site, the S/T kinase active site and a Thr–Glu–Tyr (TEY) motif. The C-terminus (blue) contains sequences involved in nuclear localization, including NLS (nuclear localization signal) and NES (nuclear export signal) sequences. Figure adapted from Fehr et al. [33]. (**B**) Consensus CDKL5 phospho-motif identified in known CDKL5 targets. (**C**) Shared properties of amino acids within the known CDKL5 phosphorylation motif demonstrate that the C-terminal residues are similar. Euler Venn diagram adapted from Taylor et al. [40].

The CDKL5 protein has a highly conserved N-terminal kinase domain consisting of an ATP-binding site, an activation site and a TEY motif activation loop (Figure 1A). CDKL5 can self-regulate kinase activity by auto-phosphorylation of the conserved MAPK TYX phosphorylation site within the kinase domain [41,42]. The archetypical feature of CDKL5 is a long C-terminal regulatory domain that contains two nuclear localization signal (NLS) and a nuclear export signal (NES, Figure 1A). CDKL5 actively shuttles between the nucleus and cytoplasm during critical stages of neuronal development, maintaining specific functions at each site [43]. The large C-terminal region is vital for the nuclear-cytoplasmic translocation [42–45], has a critical role in regulating catalytic activity and may facilitate protein binding or target specificity [38]. Although CDKL5 belongs to the CDKL family which contain putative cyclin binding domains, there is no evidence that CDKL5 specifically interacts with cyclins, and numerous bulky substitutions may prevent the binding of cyclins to CDKL5 [38].

## CDKL5 function in neurons

Human CDKL5 exists as many isoforms resulting from alternative splicing, some expressed ubiquitously [9,46] whilst some are predominant in the brain [46–48]. At the mRNA level, *CDKL5* is most abundant in forebrain structures including the cerebral cortex, hippocampus, striatum and olfactory bulb [49] and particularly abundant in forebrain neurons [50] including both glutamatergic and GABAergic neurons, with little or no expression detected in glial cells [43,51]. CDKL5-positive neurons reside in the hippocampus and cerebral cortex [43,52], and CDKL5 is distributed in both the nucleus and the cytoplasm of cortical neurons [43,44,51]. The intracellular location of CDKL5 within neurons changes throughout development, with a large proportion present in the cytoplasm at early postnatal stages, coinciding with a peak in CDKL5 expression [41,43,48,53].

Given the severe neurological phenotype of CDD, it stands to reason that CDKL5 is vitally important for normal brain development and function. *CDKL5* gene expression levels increase during neuronal maturation and during synaptogenesis [54,55] where CDKL5 concentrates in neuronal growth cones [43] and regulates actin dynamics [53], synaptic vesicle endocytosis, synaptic vesicle recycling [56], excitatory and inhibitory synaptic stability, neurite outgrowth and dendritic spine development [53,54,57]. Conversely, a lack of *CDKL5* expression results in decreased dendritic branching, impaired neuronal circuit connections [49,58], impaired synaptic vesicle recycling [56], dysmorphic dendritic protrusions [54] and a lack of higher order dendritic branches demonstrated from *in vitro* [53,59,60] and *in vivo* studies [54,61,62]. CDKL5 deficiency causes irregularity in axon formation [63] and reduces axonal length [53,64].

Several *Cdkl5* knockout animal models exist [49,58,65] that recapitulate the human disease including learning and memory impairments, autistic-like behaviors and motor deficits, and feature dysmorphic neuronal architecture, disrupted signaling pathways and impaired neuronal connectivity [20,49,54,58,60–62,66–75]. Despite extensive investigations, many *Cdkl5* animal models lack the development of spontaneous seizures characteristic of CDD [49,58] but some strains are susceptible to *N*-methyl-d-aspartate (NMDA)-induced seizures [61,65,76]. Spontaneous epileptic spasms were described in aged female *Cdkl5* mice [68], and overt, myoclonic and tonic-clonic behavioral seizure-like events were observed in aged animals (>28 weeks) [19]. The lack of these seizure-like events in homozygous females, and hemizygous males implies X-linked mosaicism may drive this phenotype in mice [19]. Using temporal manipulation of endogenous *Cdkl5* in male mice, it was demonstrated that postdevelopmental loss of CDKL5 in mice causes a similar clinical phenotype [76] to knockout *Cdkl5* animal models [20,49,58,61,66]. Excitingly, restoration of *Cdkl5* ameliorated the behavioral phenotype and aberrant NMDA receptor signaling [76], demonstrating for the first time the potential for disease reversal in CDD. The inconsistency of a seizure phenotype may be due to differences between the mouse and human brain neural network [77].

The use of CDD patient-derived iPSC provide a valuable approach to understanding the molecular and cellular functions of CDKL5 in model systems akin to the human brain [54,69,78–81] and can be used to study neural network impairments provide an alternative model system to current animal models. Human dorsal forebrain glutamatergic neurons with *CDKL5* mutations had impaired miniature excitatory postsynaptic currents, but normal inhibitory postsynaptic currents suggestive of a cell-specific phenotype in CDD [54]. CDD human cortical forebrain organoids modeled enhanced hyperexcitability compared with controls and revealed a network of proteomic and phosphoproteomic impairments [81], providing critical insight into the molecular mechanisms of CDD and proving valuable to screen for novel compounds.

## CDKL5 phosphorylation targets containing the consensus motif

A significant proportion of the human proteome is subject to phosphorylation [82,83], which acts as a rapid molecular switch, inducing conformational changes and regulating interaction with proteins and other macromolecules in the cell. Kinase dysfunction has been implicated in hundreds of human diseases ranging from cancer and inflammatory diseases through to neurological conditions. Most current kinase drug targets have been developed for non-central nervous system disorders and focus on kinase inhibition. However, there is a great interest in developing kinase-reactivating drugs in neurological conditions such as CDD. Additionally, small molecules targeting pathways perturbed by loss of CDKL5 function would also make logical drug candidates.

CDKL5 has been implicated in several essential functions within the neuron through interaction or association with other proteins, or by direct phosphorylation modification of target proteins (Table 1). The specificity of CDKL5 kinase activity is primarily determined by the consensus sequence of amino acid residues that flank the phosphorylation site of the target protein. The consensus sequence motif of CDKL5 was defined as Arg-Pro-X-Ser/Thr-Ala/Pro/Gly/Ser (Figure 1B) [69,86]. The most C-terminal amino acids within the motif share similar properties (Figure 1C) in containing small side chains that are mostly uncharged. It is possible that other residues at the C-terminal position relative to the phosphoserine are tolerated [12], although it is currently unknown whether proteins lacking this motif are direct substrates of CDKL5 [86].

**Table 1.** Proteins directly phosphorylated by CDKL5 containing the consensus CDKL5 motif

Gene	Protein	UniProt KB ID	Direct phosphorylation	Site	Motif surrounding site of consensus sequence R-P-X-[S/T]-[A/G/P/S]	Protein function in relation to CDKL5	Model system (e.g. animal, cell line, etc.)	Full length or kinase domain	*In vitro* or *in vivo*	Method of identification	Citation
ARHGEF2	Rho guanine nucleotide exchange factor 2	Q92974	+	Ser^122^	TIRE**RPSsA**IYPS	Mediates interaction between microtubules and actin cytoskeleton. Activates RHO-GTPases. Regulating dendritic spine morphology.	Mouse brain lysates, HEK293 cells	Full length and Kinase Domain	*In vitro* and *In vivo* (mouse)	Chemical-genetic approach using an ATP-analogue specific CDKL5 and identified using LC-MS/MS and western blot	[69]
AMPH1	Amphiphysin1	P49418	+	Ser^293^	PAPA**RPRsP**SQTR	Involved in neural transmission and development. Associated with clathrin-mediated endocytosis.	Mouse brain lysates, Recombinant protein from *E. coli*	Kinase domain	*In vitro*	Brain lysates incubated with CDKL5 Kinase domain with radiolabelled ATP. Mouse Amph1 purified from *E. coli* directly phosphorylated by CDKL5.	[84,85]
CEP131	Centrosomal protein of 131 kDa	Q9UPN4	+	Ser^35^	PVSR**RPGsA**ATTK	Centrosomal protein involved in the formation and function of primary cilia. Maintains centriolar satellite integrity	HEK293, U2OS	Full length	*In vitro*	Direct phosphorylation of synthetic peptide with wild-type or kinase dead CDKL5. Co-expression with CDKL5 saw increased phosphorylation	[12]
DLG5	Disks large homolog 5	Q8TDM6	+	Ser^1115^	QKRR**RPKsA**PSFR	Maintains cell polarity. Signaling to the microtubule-based cytoskeleton. Maintenance of epithelial cell structure	HEK293, U2OS	Full length	*In Vitro*	Direct phosphorylation of synthetic peptide with wild-type or kinase dead CDKL5. Co-expression with CDKL5 saw increased phosphorylation	[12]
ELOA	Elongin A	Q14241	+	Ser^311^	EENR**RPPsG**DNAR	Transcriptional elongation factor. Involved in DNA double-stranded repair	HEK293, U2OS	Full length	*In vitro*	CDKL5 overexpressed in nucleus. Targets identified via phosphoproteomic. Direct phosphorylation of synthetic peptide with wild-type or kinase dead CDKL5	[86]
EP400	EE1A-binding protein p400	Q96L91	+	Ser^729^	SPVN**RPSsA**TNKA	Involved in transcriptional activation of select genes by acetylation of nucleosome histones H4 and H2A.	HEK293, U2OS	Full length	*In vitro*	CDKL5 overexpressed in nucleus. Targets identified via phosphoproteomic. Direct phosphorylation of synthetic peptide with wild-type or kinase dead CDKL5	[86]
MAP1S	Microtubule-associated protein 1S	Q66K74	+	Ser^871^ and Ser^900^	KAPA**RPSsA**SATP and DRAS**RPLsA**RSEP	Binds to both microtubules and actin, potentially cross-linking and stabilizing the two proteins. Bridges microtubules to mitochondria. Might link NMDA receptor subunit NR3A to the cytoskeleton.	Mousse brain lysate, HEK293, U2OS	Full length and kinase domain	*In vivo* and *in vitro*	Chemical-genetic approach using an ATP-analogue specific CDKL5 and identified using LC-MS/MS and western blot. Direct phosphorylation of synthetic peptide with wild-type or kinase dead CDKL5. Co-expression with CDKL5 saw increased phosphorylation	[12,69]
EB2/MAPRE2	Microtubule-associated protein RP/EB family member 2	Q15555	+	Ser^222^	STPS**RPSsA**KRAS	Microtubule end-binding protein which regulates microtubule dynamic instability.	Mouse brain lysates, HEK293 cells	Full length and Kinase Domain	*In vitro* and *In vivo* (mouse)	Chemical-genetic approach using an ATP-analogue specific CDKL5 and identified using LC-MS/MS and western blot	[69]
TTDN1	TTD non-photosensitive 1 protein	Q8TAP9	?	Ser^40^	GGGP**RPPsP**RDGY	TTDN1 is mutated in a form of tricothiodystrophy (TTD), typically caused by failure in transcription–coupled DNA repair	HEK293, U2OS	Full length	*In vitro*	CDKL5 overexpressed in nucleus. Targets identified via phosphoproteomic methods.	[86]

A bona fide kinase substrate of CDKL5 may be considered with experimental evidence including demonstrating; (1) the target (or peptide of the substrate) can be phosphorylated by CDKL5 using *in vitro* kinase assays; (2) site-directed mutagenesis of the phosphorylation site abolishes phosphorylation by CDKL5 and (3) *in vivo* or in-cell evidence demonstrates that reduction in CDKL5 activity leads to reduction in phosphorylation, or increased activity leading to increased phosphorylation.

**Figure 2.**
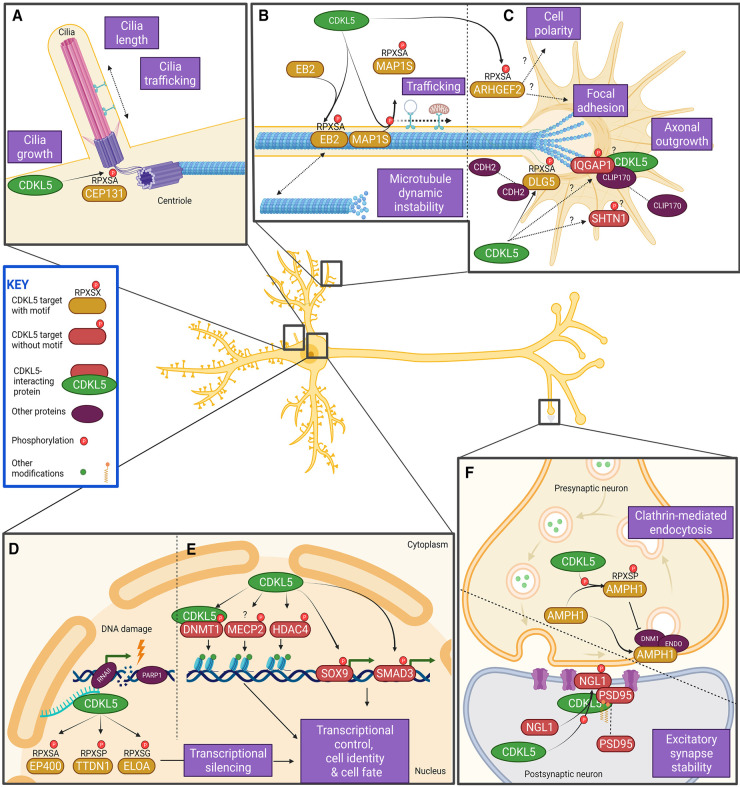
Known CDKL5 targets and affected processes in neurons. CDKL5 targets containing the consensus motif (yellow), phosphorylation targets of CDKL5 without the consensus motif or phosphorylation site not specifically determined (red) and associated proteins (purple). (**A**) CEP131 is a key component of centriolar satellites that are critical to primary cilia function among a range of critical cell functions. (**B**) MAP1S and EB2 are known direct targets of CDKL5 involved in neuronal microtubule dynamics, which may, in turn, affect microtubule dynamic instability or microtubule trafficking of cargo in neurons. (**C**) ARHGEF2 and DLG5 are both direct CDKL5 targets. ARHGEF2 can bind to microtubules and is involved in maintaining processes critical to neurons including regulation of dendritic spine morphology and focal adhesions. DLG2 controls dendritic spine formation and synaptic transmission in cortical neurons by modulating the localization of N-cadherin (CDH2). Other proteins CDKL5 may interact with or potentially phosphorylate include SHTN1, CLIP170 and IQGAP1. (**D**) DNA damage induces CDKL5 re-localization to sites of double-stranded DNA breaks (DSB). Here CDKL5 phosphorylates the direct targets ELOA, TDN1 and EP400, leading to transcriptional silencing in the region of the DSB and subsequently affects transcriptional activity. (**E**) Other potential phosphorylation targets of CDKL5 not containing the consensus motif include the chromatin remodeling proteins MeCP2, DNMT1 and HDAC4, and transcription factor SOX9 and the signal transduction protein SMAD3, all may affect transcriptional control, regulate cell identity and determine cell fate. (**F**) AMPH1 is a direct CDKL5 phosphorylation target, and phosphorylation prevents the association with endophilin (ENDO) and dynamin (DNM1), inhibiting clathrin-mediated endocytosis. This is critical for synaptic vesicle recycling, spine formation and axonal growth. There is evidence CDKL5 may phosphorylate the cell adhesion molecule NGL1, which interacts with palmitoylated PSD95 and this provides a scaffold for receptors of glutamatergic synapses. Created with BioRender.com.

We provide a summary of phosphorylation targets of CDKL5 relevant to neuronal function and CDD disease pathology (Figure 2). We provide detailed experimental evidence for each CDKL5 substrate (Table 1).

### Centrosomal protein 131 (CEP131)

CDKL5 directly phosphorylates the ciliopathy-associated protein CEP131 at Ser^35^ within the CDKL5 motif RPXS^p^A in human cell lines [12]. CEP131 regulates cellular proliferation, centriole amplification, stress-induced centriolar reorganization, chromosomal stability and DNA repair [87]. CEP131 is a component of centriolar satellites (Figure 2A) that are involved in the regulation of a complex network for primary cilia and flagella formation [88]. Phosphorylation of CEP131 plays a key role in controlling centriolar satellite status and regulating critical centrosomal functions in response to cellular stress [89]. In zebrafish, the homolog *Cep131* regulates ciliogenesis [90] and CEP131 knockout animals have phenotypes resembling human ciliopathies [90,91] primarily characterized by renal and retinal defects. Although CDD patients do not present with a classic ciliopathy disorder, primary cilia play a critical role in the developing brain [92] and their dysfunction contributes to neurodevelopmental disorders [93] and disease [94]. CDKL5 localizes to cilia, and impairs ciliogenesis when overexpressed [38]. Elongated cilia were apparent in *Cdkl5* knockout rat hippocampal neurons [95] and may be a useful phenotypic marker for high-throughput drug discovery [95].

### Microtubule-associated protein RP/EB family member 2 (EB2/MAPRE2)

EB2 is phosphorylated at Ser^222^ at the CDKL5 consensus motif of RPXS^p^A [69]. EB2 is ubiquitously expressed and regulates microtubule dynamics [96]. Human CDKL5-deficient neurons [69,80] have significantly reduced EB2 phosphorylation, and EB2 trafficking distance has been shown to be significantly increased in hippocampal neurons isolated from *Cdkl5* knockout mice [69] (Figure 2B). Interestingly, analysis of the temporal and spatial phosphorylation of EB2 by CDKL5 showed that CDKL5-directed phosphorylation of both EB2 and MAP2 peaked during early postnatal development coinciding with an increase in CDKL5 expression levels during this period [69]. EB2 phosphorylation has been shown to be predominantly in dendrites, and phosphorylation was suppressed by the NMDA receptor activity, suggesting that CDKL5 is involved in activity-dependent circuit regulation [69]. Importantly EB2 has been validated in multiple laboratories as a substrate of CDKL5 in the mouse brain, in human cell lines and iPSC-derived neurons [69,76,80,95]. The specific role that CDKL5 may have in regulating EB2 phosphorylation remains to be fully elucidated.

### Microtubule-associated protein 1S (MAP1S)

MAP1S has been identified as a direct target of CDKL5 and is phosphorylated at two positions, human Ser^871^ (mouse Ser^786^) [69] and Ser^900^ (mouse Ser^812^) [12,69]. Both phosphorylation events occur in the CDKL5 consensus motif RPXS^p^A. MAP1S has been validated as a substrate of CDKL5 in the mouse brain [56,69]. Phosphorylation of MAP1S by CDKL5 results in disassociation of MAP1S from microtubules (Figure 2B), which promotes the formation of microtubule loops at dendritic tips, required for microtubule stabilization or continued microtubule growth [69]. MAP1S is ubiquitously expressed and binds to stabilize microtubules [97], regulating this microtubule stability throughout the cell cycle [98]. MAP1S specifically bridges microtubules to mitochondria and the autophagy machinery [99]. The phosphorylation of MAP1S by CDKL5 could affect mitochondrial trafficking, which is significantly impaired in CDKL5 iPSC-derived neurons [80]. Similarly, anterograde transport and trafficking of BDNF/TrkB carrying endosomal vesicles was significantly impaired in *CDKL5* knockout neurons [69] further contributing to inefficient microtubule trafficking in CDKL5-deficient cells. MAP1S also binds to the NMDA receptor subunit NR3A [100] and may effect impaired neurotransmitter vesicle transport, which can be reversed in *Cdkl5* knockout mice by an NMDA receptor agonist [65].

### Disks large homolog 5 (DLG5)

CDKL5 phosphorylates DLG5 at Ser^1115^ at the RPXS^p^A site in human cell lines [69]. DLG5 is involved in maintaining cell polarity, cell adhesion, proliferation and transmission of extracellular signals to the cytoskeleton and membrane [101,102]. Additionally, DLG5 regulates synaptogenesis, dendritic spine formation and synaptic transmission in cortical neurons by modulating the subcellular localization of N-cadherin (CDH2, Figure 2C) [102]. Interestingly, *DLG5* variants are associated with ciliopathy-like phenotypes and congenital anomalies, and depletion of *Xenopus dlg5* disrupts brain ventricle and kidney morphology [103]. Despite this, the precise effect of CDKL5 phosphorylation on DLG5 remains unexplored.

### Rho-Rac guanine nucleotide exchange factor 2 (ARHGEF2)

ARHGEF2 is phosphorylated by CDKL5 at Ser^122^ in the consensus motif of RPXS^p^A [69]. ARHGEF2 is a guanine exchange factor (GEF), which facilitates the exchange of GDP for GTP on Rho GTPases. Although over 80 GEFs have been identified, however, only a handful are associated with the microtubule network. ARHGEF2 is partly regulated by the microtubule polymerization state, and by serine and tyrosine phosphorylation. ARHGEF2 can bind directly to microtubules and is involved in the regulation of numerous processes. These include dendritic spine morphology, focal adhesions, cell motility, regulation of polarization during the cell cycle regulation and epithelial barrier permeability (Figure 2C) [104]. Disruption of these processes has been well-recorded in CDKL5-deficient cells (as discussed above), and disrupted phosphorylation of ARHGEF2 may explain some of these processes. Despite this, the specific effects of ARHGEF2 phosphorylation by CDKL5 have yet to be fully determined, and multiple kinases are known to phosphorylate ARHGEF2 [105].

### Elongin A (ELOA)

Recently it was shown that in response to DNA damage, CDKL5 is recruited to phosphorylate ELOA, TTDN1 and EP400 (Figure 2D) [86]. ELOA is phosphorylated at Ser^311^ within the consensus CDKL5 phosphorylation site of RPXS^p^G and has been confirmed as a direct target of CDKL5 in human cell lines [106]. ELOA has a tightly regulated dual role both in resting cells as a component of the Elongin complex that stimulates the rate of RNA polymerase II (Pol II) elongation, and in cells under transcriptional stress, where it acts as a subunit of the Cullin-RING (CRL) E3 ubiquitin ligase, which targets Pol II that is stalled at double-strand breaks (DSBs) [107]. ELOA likely ubiquitinates a range of proteins at DNA damage sites. Transcriptional silencing at DSB is a common response to allow access to DNA repair proteins and to reset the chromatin status by enhancing access of the damage repair machinery to DSB [108]. Physiological brain activity causes DSBs to occur in neurons, and this has been reported in multiple brain regions of wild-type mice [109]. DSBs are particularly pronounced in the dentate gyrus, involved in learning and memory and DSBs are also increased by stimulation [109]. CDKL5-deficient neurons and brains have been shown to have increased DNA repair protein levels [106], elevated levels of DNA damage and higher apoptosis rates [110].

CDKL5 may play a critical role in identifying DNA damage in neuronal cells and failure of these processes would have significant consequences for post-mitotic neurons. Human neuroblastoma cells with a *CDKL5* deletion are hypersensitive to DNA-damage induced stress and showed DNA damage-associated markers (γH2Ax, RAD50 and PARP1), reduced cell viability and impaired neuronal maturation [110]. CDKL5 is recruited to sites of DNA damage and initiates the silencing of genes harboring DNA breaks [86]. The recruitment of CDKL5 and ELOA requires both the local synthesis of poly(ADP-ribose) (PAR), which mediates chromatin relaxation, and active transcription, leading to transcriptional silencing of genes close to, or at the site of double-stranded DNA breaks (Figure 2D). The C-terminal region of CDKL5 binds to PAR after DNA damage [86]; however, it is unclear whether this interaction affects CDKL5 catalytic activity. Although recruitment of ELOA to DNA damage sites was not dependent on Ser^311^ phosphorylation, the functional significance of this phosphorylation event remains undetermined.

### Trichothiodystrophy non-photosensitive 1 (TTDN1)

TTDN1 is a CDKL5 target in human cell lines and is phosphorylated in response to DNA damage [86]. TTDN1 is phosphorylated by CDKL5 at Ser^40^ in the consensus sequence RPXS^p^P. Although little is known of TTDN1 function, it has a potential role in the maintenance of cell cycle integrity by regulating mitosis or cytokinesis (Figure 2D). Pathogenic variants in *TTDN1* cause trichothiodystrophy (TTD), a rare multisystem disorder caused by genes involved in DNA repair and transcription. TTD has some features similar to CDD, including seizures and developmental delay [111]. The significance of this phosphorylation event remains to be determined.

### E1a binding protein P400 (EP400)

EP400 was identified in human cell lines following a screen for nuclear CDKL5 targets responding to DNA damage [86]. EP400 is phosphorylated at Ser^729^ at the consensus sequence RPXS^p^A. EP400 is a chromatin remodeling protein [112], and is a component of the Nu4A histone acetyltransferase that regulates the expression of specific genes by acetylation at specific genomic regions (Figure 2D) [113]. Further studies are required to elucidate the significance of EP400 phosphorylation.

### Amphiphysin 1 (AMPH1)

CDKL5 directly phosphorylates AMPH1 [84] at Ser^293^ in the consensus CDKL5 motif RPXS^p^P [85]. Phosphorylation of AMPH1 prevents the association with endophilin (ENDO) and dynamin (DNM) which suppresses clathrin-mediated endocytosis (Figure 2F). Endocytosis is critical to synaptic vesicle recycling, spine formation and axonal growth, both processes impaired in CDKL5-deficient neurons [53,54,57]. The phosphorylation of AMPH1 by CDKL5 may influence neuronal activity at the synapse, but recent evidence demonstrates that AMPH1 is phosphorylated independent of CDKL5 at the synapse [56].

## Putative substrates and CDKL5-interacting proteins provide molecular insight to CDKL5 function

Although there is a rapidly advancing knowledge of direct phosphorylation targets containing the consensus motif recognized by CDKL5, there are many instances of proteins reported to be phosphorylated by CDKL5 that do not contain the known CDKL5 consensus motif (Table 2). Additionally, the long, largely unstructured C-terminal tail of CDKL5 may aid direct interaction with proteins, which may bring proteins within proximity to CDKL5 for phosphorylation, despite the absence of the consensus motif (Table 2). Some caution is required when assessing proteins lacking the CDKL5 consensus motif as to whether these proteins are considered bona fide phosphorylation targets of CDKL5. Careful assessment of experimental evidence is required on a case-by-case basis and is summarized in Table 2. Nevertheless, the study of these proteins provides unique insights into the molecular function of CDKL5, and further research may validate or negate the direct phosphorylation by CDKL5.

**Table 2.** Proteins phosphorylated by CDKL5 but do not contain the consensus CDKL5 motif

Gene	Protein	UniProt KB ID	Interaction	Direct phosphorylation	Site	Motif surrounding site	Protein function of target	Model system (eg animal, cell line, etc.)	Full length or kinase domain	*In vitro* or *in vivo*	Method of identification	Citation
DNMT1	DNA methyltransferase 1	P26358	+	+	?	Site not reported	Maintains patterns of methylated cytosine residues. It should be noted that the phosphorylation of Dnmt1(1–290) by CDKL5 was significantly stimulated in the presence of DNA. MeCP2 is also a well-known DNMT1-binding protein.	Mouse brain extract, HEK293T, Recombinant protein from *E. coli*	Full length and Kinase domain	*In vitro*	Nuclear co-localization studies in HEK293T cells. GST pulldown assays from mouse brain extract and HEK239T cells. Mouse DNMT1 purified from *E. coli* directly phosphorylated by CDKL5.	[114]
HDAC4	Histone deacetylase 4	P56524	+	+	Ser^632^	RPLSRAQsSPASAtF	An enzyme that catalyzes histone de-acetylation, repressing transcription. Key role in brain development and neuronal survival. In neurons, primarily in cytoplasm but upon CDKL5 loss, translocates to the nucleus reducing histone acetylation.	KO mice, HEK293T, SH-SY5Y	Full length	*In vitro* and *In vivo*	Phosphoprotein profiling of SH-SY5S cells by the Phospho Explorer antibody microarray. Confirmed targets by co-immunoprecipitation and direct phosphorylation studies *in vitro* and *in vivo*.	[115]
NGL-1, KIAA1580, LRRC4C	Netrin-G ligand-1	Q9HCJ2	+	+	Ser^631^	PLLIRMNsKDNVQET	Transmembrane protein highly expressed in the brain which interacts with a specific axon guidance molecule. Axon guidance molecule	Mouse brain extract, HEK293T, COS-7, Primary fibroblast, iPSC-derived neurons	Full length	*In vitro* and *In vivo*	Co-immunoprecipitation *in vitro* and *in vivo*, co-localization studies in neurons and western blotting. Phosphorylation confirmed in overexpression studies and direct phosphorylation of peptide incubated with CDKL5.	[54]
SMAD3	Mothers against decapentaplegic homolog 3	P84022	+	+	?	Site not reported	Functions within the TGFβ pathway, regulating transcription by transducing signals from the cell membrane to the nucleus.	ouse brain extract, HEK293T, SH-SY5Y, primary mouse neurons	Full length	*In vitro* and *In vivo*	Phosphoprotein profiling of cortex extracts from CDKL5 wt and KO mouse cells by the Phospho Explorer antibody microarray. Co-immunoprecipitation assays in established cell lines. Expression analysis in CDKL5 KO mouse neurons and direct phosphorylation confirmed with purified recombinant protein from HEK293T	[52]
SOX9	Transcription factor SOX-9	P48436	+	+	Ser^199^	ATEQTHIsPNAIFKA	Transcription factor of the SOX family with critical roles in cell fate determination. Sox9 suppresses cell death during development, adult tissue homeostasis, and oncogenesis. Phosphorylation-dependent suppression of pro-survival transcription regulator Sox9	Mouse renal tissue, HEK293	Full length	*In vitro* and *In vivo*	Identified using siRNA Kinomic screening and direct phosphorylation confirmed with recombinant human protein and in mouse renal tissue extract. CDKL5 physically interacts with SOX9 as shown by co-immunoprecipitation.	[116]
IQGAP1	IQ Motif Containing GTPase Activating Protein 1	P46940	+	?	?	Site not reported	IQGAP — Fundamental regulator of cell migration and polarity by regulating cytoskeletal effector proteins	Primary mouse neurons, HeLa, COS-7	Full length	*In vitro*	Interaction by yeast-two hybrid screening and immunoprecipitation from mouse brain lysates. No direct phosphorylation shown, however, IQGAP localization in CDLK5 KO cells could be rescued with WT CDLK5 but not Kinase dead mutant.	[59]
MeCP2	Methyl-CpG binding protein 2	P51608	+	?	?	Site not reported	Binds to methylated DNA and represses transcription. Rett syndrome gene. Can activate and repress transcription	HEK293T	Full Length	*In vitro*	GFP pulldown assay in HEK293T. Purified recombinant protein incubated with CDKL5 showed phosphorylation. Follow up *in vitro* kinase assays contradict MeCP2 as a CDKL5 substrate.	[41,42]
PSD95, DLG4	Postsynaptic density protein 95	P78352	+	?	NA	Site not reported	PSD95 is critical for dendritic spine development	Rat brain extract, HEK293T	Full length	*In vitro*	GST pulldown with recombinant PSD95 and CDKL5 in HEK293T. Affinity purification of endogenous PSD95 from rat brain extract using column bound CDKL5	[57]
SHTN1, SHOT1	Shootin1	A0MZ66	+	?	?	Site not reported	Promotes neuron polarization and axon outgrowth.	Mouse brain extract, primary mouse neurons	Full length	*In vitro*	Yeast two-hybrid screening identified SHTN1. Confirmed with Co-immunoprecipitation of endogenous SHTN1 and CDKL5 in brain lysates. No direct phosphorylation shown however, SHTN1 phosphorylation levels reduced in CDKL5 KO neurons.	[63]

### Axon and dendrite-remodeling proteins (IQGAP1 and SHTN1)

Neuronal morphogenesis defects can be caused by dysregulation of the cellular cytoskeleton, which is critical for many fundamental cellular processes including neural migration, neurite formation and long-range intracellular transport of cargo to dendrites and synapses. CDKL5 is highly expressed in areas rich in actin [53], a key structural component of the cytoskeleton [67], and has a strong association with microtubules [117] and microtubule-associated proteins [12,64,69]. CDKL5 may be involved in cytoskeletal-regulated events such as cell migration [59] and cell division [64].

CDKL5 is associated with the actin cytoskeleton and interacts with IQ motif containing GTPase activating protein 1 (IQGAP1; Figure 2C) [59]. This interaction is required for IQGAP to form a functional complex with activated GTPases Rac1 [118] and Cdc42 via the microtubule plus-end protein cytoplasmic linker protein 170 (CLIP170) [119] which together regulate dendritic morphology [120]. Disruption of this interaction causes disassociation of the microtubule plus-end tracking protein (+TIP) CLIP170, resulting in deranged microtubule dynamics [59]. It is unclear whether CDKL5 may directly phosphorylate IQGAP1, nevertheless serine phosphorylation at S1443 opens the IQGAP1 structure, allowing binding of IQGAP1 to the downstream effector proteins Cdc42 [121], N-WASP, CLIP170 and the exocyst complex [122]. It has been speculated that CDKL5 acts as a kinase scaffold protein for the MAPK kinase signaling pathway involved in synaptic plasticity and memory [123].

CDKL5 is a shootin1 (SHTN1)-interacting protein identified using yeast-two hybrid screening, with this interaction confirmed *in vivo* [63]. SHTN1 has a well-established role in axon formation during neuronal polarization, partly regulated by the interaction with CDKL5 (Figure 2C). SHTN1 phosphorylation transduces chemical signals into traction forces to facilitate axonal outgrowth [63]. Although it is unclear whether CDKL5 directly phosphorylates SHTN1, and which residues are modified, there is evidence of decreased SHTN1 phosphorylation in neurons with reduced CDKL5 expression.

### Synaptic adhesion and scaffolding proteins (NGL-1 and PSD-95)

CDKL5 physically interacts with, and phosphorylates, the synaptic adhesion molecule netrin-G ligand-1 (NGL-1) at Ser^631^ (Figure 2F), but not within the CDKL5 consensus motif. Phosphorylation of NGL-1 ensures a stable association with the scaffold protein Postsynaptic density protein 95 (PSD-95). CDKL5 also binds to PSD-95, through the C-terminal tail, with binding governed by palmitoylation of PSD-95 [54,57]. This ensures that PSD95 is targeted to newly forming dendritic protrusions and excitatory synapses [57]. Here, NGL-1 and PSD95 serve as a scaffold for AMPA-type glutamate receptors, a key component of glutamatergic synapses [124]. This process is critical for normal neuronal spine development, a process that is significantly impaired in CDKL5-deficient neurons. Mutation of the Ser^631^ phosphorylation site prevents NGL-1 from maintaining synaptic contacts [54], which may explain dendritic spine instability and derangement in CDKL5-deficient neurons.

### Chromatin remodeling proteins: MeCP2, HDAC4 and DNMT1

CDKL5 promotes the proliferation and differentiation of neuronal cells by regulating cell cycle progression [55], and knockdown of CDKL5 by RNAi causes multipolar spindle formation, centrosome accumulation and failure of cytokinesis [64]. Interestingly, in the *Cdkl5* knockout mice there is increased apoptosis of post-mitotic granule neuron progenitors and severe dendritic hypotrophy [60]. CDKL5 co-localizes with nuclear speckles, enriched in pre-mRNA splicing factors, and may regulate the dynamic activity of nuclear speckles [125]. In the nucleus, CDKL5 directly interacts with and may phosphorylate several proteins that regulate chromatin remodeling and DNA methylation (Figure 2E), including methyl-CpG binding protein 2 (MeCP2) [41,44,46], histone deacetylase 4 (HDAC4) [115] and DNA methyltransferase 1 (DNMT1) [114]. Both MeCP2 [126] and CDKL5 associate with centrosomes and at the midbody of dividing cells [64], which acts as the main microtubule organizing center and regulator of cell cycle progression. The loss of centrosomal activity arrests cells in premature senescence at the G1-S transition phase. Since centrosomal activity is regulated by several kinases, it is reasonable to predict that CDKL5 may in part regulate centrosomal activity and cell cycle regulation effecting both neural progenitor proliferation and differentiation.

### Transcription factors (SOX9 and SMAD3)

Increasing evidence highlights CDKL5 as kinase involved in stress response. Recently, a potential non-neuronal function of CDKL5 was identified in a kinome-wide screen in renal tubular epithelial cells. CDKL5 was found to phosphorylate the transcription factor SRY-box transcription factor 9 (SOX9; Figure 2E) and suppress the pro-survival transcriptional activity of SOX9, resulting in renal injury [116]. Although phosphorylation of SOX9 at Ser^199^ is not within the CDKL5 consensus motif, this phosphorylation event reduced the stability of the SOX9 protein. Although the role of SOX9-mediated phosphorylation by CDKL5 has not yet been explored in neurons, phosphorylation modification of SOX9 has a critical role in neural crest delamination [127] which is an essential step for the subsequent migration and differentiation of neural crest cells, and ultimately, cell fate determination in the developing central and enteric nervous system.

Another identified phosphorylation target of CDKL5 is the transcriptional factor SMAD3, although the exact phosphorylation site remains undetermined. Direct phosphorylation of SMAD3 protein by CDKL5 promotes SMAD3 stability, conversely, reduced SMAD3 levels impaired neuronal survival and maturation [52]. This observation may be relevant to CDKL5 pathology since defective cell survival is reported in CDD [52,60,110]. SMAD3 signaling integrates transforming growth factor β (TGF-β) signaling with cell-type specific and essential signaling pathways (Figure 2E). Interestingly, co-treatment with TGF-β normalizes SMAD3 levels and increases the survival of *Cdkl5* KO neurons, providing evidence for a potential new therapeutic target for CDD [52].

## Conclusion

The compromised ability of specific kinases to regulate intracellular signaling networks commonly underlies disease pathogenesis, particularly in neurological conditions. However, understanding the disrupted signaling pathways poses a fundamental challenge in neurobiology. Therefore, it is essential we understand the molecular pathways and the phosphorylation targets of CDKL5 and how this relates to CDD pathophysiology, progression and disease sub-types. In-depth knowledge of molecular pathways regulated by CDKL5 may reveal druggable targets which could be exploited to fast-track the development of targeted therapeutics that address directly, the molecular cause of CDD. We anticipate that in addition to directly benefitting patients with pathogenic *CDKL5* variants, understanding CDKL5 signaling pathways will have broad implications for understanding neurodegenerative disorders, in particular the fundamental importance of kinase-regulated signaling pathways in the brain. By translating and implementing such fundamental scientific research findings, an in-depth knowledge will have the power to rapidly deliver new therapeutics to transform the health of children who would otherwise suffer a devastating progressive disease trajectory because of CDKL5 deficiency.

## Perspectives

- CDD is a serious health condition, current treatment and existing therapies target disease symptoms, rather than addressing the underlying abnormal biological processes. There is an urgent need to advance our understanding of CDD biology so that we can test therapies that might significantly alter the CDD disease trajectory in young children.
- Whilst the progressive nature of CDD provides an excellent opportunity for disease intervention, without an intimate knowledge of CDKL5 function and phosphorylation targets we cannot effectively develop new effective therapeutics. By understanding the cellular targets of CDKL5, we can direct the development of therapeutics specifically towards these targets. This is necessary before significant progress can be made in the development of novel targeted therapies.
- The identification of new CDKL5 targets that modulate aberrant neuronal activity in CDD will likely have broader implications for other severe neurological disorders.

## Abbreviations

AMPH1: Amphysin1
ARHGEF2: Rho-Rac guanine nucleotide exchange factor 2
BDNF: brain-derived neurotrophic factor
CDD: CDKL5 deficiency disorder
CDH2: N-cadherin
CDKL5: cyclin-dependent kinase-like 5
CEP131: centrosomal protein 131
CLIP170: cytoplasmic linker protein 170
CMGC: cyclin-dependent kinase (CDK), mitogen-activated protein kinase (MAPK), glycogen synthase kinase (GSK3), CDC-like kinase (CLK) kinase family
DLG5: disks large homolog 5
DNM: dynamin
DNMT1: DNA methyltransferase 1
DSB: double-strand break
EB2: microtubule-associated protein RP/EB family member 2
ELOA: elongin A
ENDO: endophilin
EP400: E1A binding protein P400
GEF: guanine exchange factor
HDAC4: histone deacetylase 4
iPSC: induced pluripotent stem cell
IQGAP1: IQ motif containing GTPase activating protein 1
KO: knockout
MAP1S: microtubule-associated protein 1S
MAPK: mitogen-activated protein kinase
MeCP2: methyl-CpG Binding Protein 2
NES: nuclear export signal
NGL-1: netrin-G ligand-1
NLS: nuclear locatization sequence
NMDA: *N*-methyl-d-aspartate
PSD-95: postsynaptic density protein 95
RTT: Rett syndrome
SHTN1: shootin1
SMAD3: mothers against decapentaplegic homolog 3
SOX9: SRY-box transcription factor 9
TGF-β: transforming growth factor β
TIP+: microtubule plus-end tracking protein
TTD: trichothiodystrophy
TTDN1: trichothiodystrophy non-photosensitive 1
XCI: X-chromosome inactivation
